# Indigenous and Traditional Visual Artistic Practices: Implications for Art Therapy Clinical Practice and Research

**DOI:** 10.3389/fpsyg.2020.01320

**Published:** 2020-06-16

**Authors:** Girija Kaimal, Asli Arslanbek

**Affiliations:** Department of Creative Arts Therapies, College of Nursing and Health Professions, Drexel University, Philadelphia, PA, United States

**Keywords:** indigenous, traditional, healing, well-being, art therapy

## Abstract

In this paper, we present a review of research on the role of traditional and indigenous forms of visual artistic practice in promoting physical health and psychosocial well-being, particularly as it relates to the discipline of art therapy. Using extant literature we present an overview of how art making has historically had a therapeutic role in human lives and how it can inform the modern interpretation and profession of art therapy. Thereafter, we provide a critical review of specific studies that reference traditional and indigenous art forms in art therapy in order to invite discussion, dialogue, and awareness of the role of the arts in human development and the therapeutic role of the arts. Gaps in research areas for further study are proposed. Implications for clinical practice including expanding the scope of traditional forms of creative self-expression and promoting an informed and respectful understanding of the role of these artforms in the profession of art therapy worldwide, are also discussed.

## Introduction

Artistic expression has historically and simultaneously evolved as a part of human civilizations around the world (Dutton, 2010). Many scholars assert that art making is an integral part of human functioning and would not have evolved or been a sustained part of human existence if it did not serve a significant adaptive purpose (Dutton, 2010; Davies, 2012; Kaimal, 2019). These scholars argue that art and art making have identified roles in evoking emotions, problem solving, imagination, memorializing life events, and enabling non-verbal communication (Dissanayake, 2000; Dutton, 2010; Winner, 2018). Dissanayake (2000) for example, identified art making as being integral to humans in establishing rituals and communication as well as, in “making special” key events and milestones in the lives of individuals in communities. There has, however, been limited examination of the therapeutic role of the arts in helping individuals cope in response to challenges including deriving strength from individual creative capacity and sense of community.

In the present day, mental health issues are a worldwide challenge: The World Health Organization (2019) cautioned that lack of mental well-being is a leading cause of disease burden around the world, with one-fourth of the global population at risk of developing a mental health challenge in their lifetime and one-fifth of children and adolescents having mental health problems. Wars, adversity, discrimination, illness, and natural disasters further exacerbate these unmet needs of psychosocial support. Most parts of the world have very limited resources and few skilled professionals to support mental health and well-being.

Building on the idea of the universal presence of artistic practices and the mental health needs of our modern world, therapeutic approaches in art therapy have been theorized to be embedded in our evolutionary survival instincts including responding to threats and making choices to ensure safety and belonging (Kaimal, 2019). Facilitation of creative visual self-expression to promote mental health and overall well-being is the goal of professional art therapists. The modern profession of art therapy originated in the United States and Europe in the twentieth century as a restorative practice that allowed for self-expression in non-verbal ways like drawing and painting. In particular, the profession began by serving the needs of veterans from the two World Wars who were suffering from post-traumatic stress and to address the development of children with special needs (Junge, 2010). The American Art Therapy Association currently defines art therapy as an integrative mental health and human services profession that enriches the lives of individuals, families, and communities through active art making, creative processes, applied psychological theory, and human experience within a psychotherapeutic relationship (American Art Therapy Association, 2018). Given its origins, art therapy has been a very Western field and has been critiqued for its lack of diversity and limited understanding of the range of artistic practices around the world (Joseph, 2006; Moon, 2010; Talwar, 2010). Recent scholarly and artistic endeavors are beginning to explore indigenous knowledge and insight developed through generations of observations and deep understanding of nature and natural processes (Langlois, 2018; Whiting, 2018). There is increasing awareness of the value of traditional healing practices, including narrative storytelling (Gotschall, 2013; Duff, 2018) and integrating cultural practices like sweat lodges in medical hospitals (Center for Mental Health, 2019). Huotilainen et al. (2018) highlighted the role of arts and crafts in building community and feelings of wellness through flow states (Csikszentmihalyi, 1990) induced during art making. James (2019) further highlighted the return to traditional artistic practices like embroidery as a means to cope, for example, among migrant women facing adversity and despair.

At the same time, it is important to recognize how knowledge about human development and the conceptualizations of traditional art making relate to contemporary society and an understanding of therapeutic art making and arts for health and well-being (Pascoe, 2015). Although, knowledge is also limited about the role specifically of visual expressive traditions in health and well-being, there is a need to help develop approaches to diversity in art therapy practice and reduce the likelihood of culturally misinformed or ill-suited Western imperialist approaches to treatment (Kleinman, 1981; Whitaker, 2002). There is a need to be better informed such that cultural forms of expression are not appropriated incorrectly; efforts to use them are respectful of local cultural and spiritual traditions; and; are adapted into materials, media, and art therapy approaches as sensitively and ethically as possible (Fincher, 1991; Kaimal, 2015; Iyer, 2018). This indigenous knowledge (Chilisa, 2012; Pascoe, 2015) highlights the importance of traditional wisdom and insight, including the close interaction of artistic practice with natural materials, creative agency, and contemplative and spiritual meaning making (Franklin, 2017) while recognizing our interconnectedness with other living beings in nature (Pascoe, 2015; Nagarajan, 2018).

The purpose of this mini review paper is to invite critical review, discussion, dialogue, and awareness on the therapeutic role of the arts, in order to expand the scope of traditional approaches to creative expression and promote an understanding of the role of integrating the arts and the profession of art therapy worldwide with traditional knowledge and wisdom. Inspired by the authors’ own artistic heritage from India and Turkey, we sought to better understand the existing literature on the use of traditional and indigenous artforms in art therapy practice.

## Approaches in Art Therapy Using Traditional and Indigenous Art Forms

The databases searched for literature review included PubMed, JSTOR Google Scholar, and Elsevier Science Direct. Search terms used were, “indigenous,” “traditional,” “art therapy,” “creativity,” and “healing.”Google scholar provided the most references. We didn’t limit the year of publication but the language of source was limited to English. The search yielded nine peer-reviewed articles that referenced art therapy and indigenous arts practices. Book chapters were not included in this review. The nine papers included theoretical and critical perspectives (Cameron, 2010; Weinberg, 2018; Napoli, 2019) as well as community and ethnographic methodologies (Campanelli, 1996; Huss and Cwikel, 2005; Basto et al., 2012; Lu and Yuen, 2012; Warson, 2012; Warson et al., 2013). A brief overview of these art therapy articles is included in Table 1 and also summarized below.

**TABLE 1:** Peer reviewed journal articles addressing indigenous and traditional art practices in the discipline of art therapy.

**Article title**	**Authors (year)**	**Specific indigenous culture**	**Methodology**	**Data sources**	**Main findings**
Using the arts as a therapeutic tool for counseling–an Australian Aboriginal perspective	Cameron, 2010	Australian Aboriginal communities	Critical literature review	Art therapy books and published articles	The arts provide a culturally safe environment that connects different communities and can be a culturally appropriate mental health tool for practitioners to be connected with the indigenous people. Cultural and communication misunderstandings between indigenous and non-indigenous people are a barrier in healthcare.
Ethical contemporary art therapy: honoring an American Indian perspective	Napoli, 2019	Native American	Perspective paper with a critical literature review	Author’s views, experiences, art therapy books, and published articles	Given the United States’ position within the historical context of colonization and silencing of Native perspectives, the author identifies the first steps for contemporary art therapy working with Native American communities as that of building trust. Art therapists are encouraged to also constantly question cultural appropriation, cultural genocide, and colonial amnesia within the field of art therapy.
Gaining cultural competence through alliances in art therapy with indigenous clients	Weinberg, 2018	Indigenous peoples of Canada	Critical literature review	Art therapy books and published articles	Art therapists working with indigenous communities should invest in extensive experiences with the indigenous culture to build trust and create safety. Art therapists cannot presume cultural competence when they are working with indigenous communities.
Exploring American Indian adolescents’ needs through a community-driven study	Basto et al., 2012	Coharie and Lumbee Nation (Native American tribes)	Ethnographic study	Description of community engagement and artmaking, interviews with participants	Tribal identity is a source of pride and some participants felt that this pride should be reinforced. Participants often referred to the need for community involvement research. The authors highlights the need for and recognition of American Indian communication styles was limited, including its distinct structure and organization of verbal responses.
Pioneering in Perth: art therapy in Western Australia	Campanelli, 1996	Australian Aboriginal communities	Description of art therapy program development that aligns with Aboriginal values	Personal art therapy practice experiences and observation	Three examples of successful collaborations between artists and art therapists working with Aboriginal communities around Perth, Australia. The examples highlight the values around artmaking including the focus on community well-being and the related distrust of individually focused White models of therapy.
Researching creations: applying arts-based research to Bedouin women’s drawings	Huss and Cwikel, 2005	Israeli Bedouin community	Arts based research	Artworks, author notes, and observations	Art as therapy or empowerment can offer transformative, enriching and empowering elements of creating art, making it a worthwhile endeavor for the women. Art research can enhance understanding between the Bedouin women and the dominant Israeli culture by offering a complex multifaceted experience of Bedouin women’s concerns.
Journey women: art therapy in a decolonizing framework of practice.	Lu and Yuen, 2012	Inuit community (Aboriginal and non-Aboriginal heritage)	Qualitative analysis of a workshop integrating a decolonizing framework of art therapy and aboriginal artistic practices	Artworks, author notes, and observations	This (research) workshop incorporated indigenous ceremonies such as smudging, drumming, singing and movement, poetry and art creation with art directive focused on body mapping. The public exhibition of artwork from the workshop contributed to the goals the empowerment and awareness building on discrimination faced by indigenous women.
Healing pathways: art therapy for American Indian cancer survivors	Warson, 2012	American Indian and Alaska Native	Mixed-Method – Single-group pretest and posttest with a qualitative content analysis of the artwork	State-Trait Personality Inventory (STPI) and artwork	The design builds a culturally relevant workshop format which can be used in a larger study. The results of this study reinforce a Native-American concept of wellness-based mind, body, spirit, and context. No statistically significant relationship was found as a result of the intervention. Some of the lack of change was attributed to the inability of standardized measures to accurately address the culturally diverse experiences of participants.
Healing pathways: American Indian medicine and art therapy	Warson et al., 2013	Coharie Native American tribe	Co-created ethnographic study of wellness practices for the Coharie community	Art images and narrative responses of the participants	Complementary forms of therapy, particularly art therapy and forms of story-sharing served as a community-building project as well as a method to voice practices larger community settings. The most desired wellness strategy was identified as mindful awareness through sensory integration while the least effective was found to be lack of awareness of holistic practices among Cohorie youth.

The three theoretical perspective papers highlighted the differences in understanding of healing between the indigenous and Western societies (Cameron, 2010; Weinberg, 2018; Napoli, 2019). Cameron (2010) discussed how indigenous healing was informed by holistic understanding of the individual, including the “social, physical, mental, emotional, spiritual, and cultural states of being” (Cameron, 2010, p. 404). Cameron (2010) further states that indigenous Australians consider art as a daily practice of meaning making rather than an artistic ideal. Further referencing communication difficulties and cultural misunderstandings that can occur between the art therapist and the indigenous client, Weinberg (2018) highlighted the importance of cultural appropriateness in art therapy when working with indigenous clients. Reflecting on the historical mistrust of indigenous people toward non-indigenous health-care providers, Weinberg (2018) suggested that art therapists consider cultural barriers and recommended partnering with indigenous mentors when practicing in an indigenous-focused art therapy program. In line with this argument, Cameron (2010) also underlined that utilizing indigenous artistic expressions in therapy acknowledges cultural inclusion and aids cross-cultural communication. When serving indigenous communities, the non-indigenous therapist should gain cultural competency, learn about trans-generational history, recognize social values and create culturally safe spaces in order to provide best practices (Cameron, 2010; Weinberg, 2018). In a strong critique of misappropriation of healing traditions, Napoli (2019) draws attention to the disrespect of American Indian traditions and practices and misuse of Native American healing systems in art therapy. For example, Napoli (2019) references the misappropriation of Native American spiritual and shamanistic practices in art therapy (McNiff, 1981). She highlights the need for culturally informed and respectful practices.

In addition to the perspective papers above, six papers used qualitative, participatory, mixed methods, and ethnographic methodologies to examine the role of art therapy practices in indigenous communities in Australia, Canada, Israel and the United States.

Campanelli (1996), underlined the importance of arts engagement for cultural restoration for Aboriginal health and well-being in Australia. His clinical work in building a community-based art therapy program in Perth, the capital city of Western Australia, highlighted the functions of Aboriginal art in cultural healing, including community involvement, cultural restoration, and recovery, as well as maintaining hope and wholeness (Campanelli, 1996). Indigenous individuals referenced taking cultural pride in being members of their community and reproducing symbols of this belonging through art making which further strengthened their sense of belonging (Campanelli, 1996; Basto et al., 2012). Campanelli (1996) highlights in particular the distrust of individualistic White approaches to health which might be perceived as a an extension of the history of oppression of aboriginal peoples in Australia, but that respectful engagement through art could be the bridge. Lu and Yuen (2012) reported on a women’s program that they developed in Canada for First Nation, Inuit, and Métis women using a decolonizing framework of practice, in order to “empower and inform people in their healing journey” (Lu and Yuen, 2012, p. 192). The authors introduced a body-mapping technique that combined art therapy with traditional practices such as drumming, prayers and smudging and in collaboration with the community refer to the research efforts as a “ceremonial event” (p. 193). Based on interviews with the participants, the authors concluded that the initiative resulted in increased feelings of empowerment, creativity, and collective strength among the participants (Lu and Yuen, 2012).

In Israel, Huss and Cwikel (2005) examined the role of art therapy and arts-based research when working with Bedouin women living in Israel. Through the use of visual data gathering tools (paper, oil pastels, color pencils, and wet paint) of arts based research inquiry, they reported that art making was a “worthwhile tool for empowerment, that can transform, enrich, and empower” the Bedouin women participants in their study (Huss and Cwikel, 2005, p. 59).

In the US, three studies examined the role of art making as a medium for community building through addressing cultural practices American Indian Coharie people. Warson et al. (2013) conducted a qualitative ethnographic study with the Coharie people in the US to emphasize the importance of a culturally relevant co-created workshop format for restoring traditional wellness approaches. The authors used a qualitative ethnographic model, incorporating community participation and recruited 44 members from the Coharie tribe. The art workshops combined with holistic healing practices provided a community building opportunity as well as a space to voice cultural practices that were not previously discussed. Another qualitative ethnographic study with Coharie adolescents highlighted that art workshops increased community involvement and was a protective factor for American Indian youth (Basto et al., 2012). Lastly, Warson (2012) study explored the impact of culturally aligned art-based interventions on stress reduction for Native American cancer patients. Her research also provided an example for cultural considerations in assessment procedures. She utilized the State-Trait Personality Inventory (STPI) which is an assessment drawn from STAI assessment, a widely used and validated measure for Western participants. However, even after several modifications to make the inventory more culturally applicable, the authors reported that inventory was found to not be suitable for the Coharie tribe, highlighting the limitations of using some standardized psychological measures in this context.

In summary, the few studies in art therapy reviewed above highlight the opportunities for empowerment and community building as well as the challenges of working from the Western perspectives of individual clinical diagnostic categories in art therapy research and clinical practices.

## Discussion

In this paper we provided a systematic review peer-reviewed literature on how art therapists have integrated traditional and indigenous artforms into their practice as well as preliminary efforts to conduct research on the topic. Overall the literature indicates that the therapeutic aspects of indigenous forms for visual arts practice are deeply interconnected with spiritual traditions, narrative storytelling, counseling and wisdom for the community, respect of all things living and non-living in nature, using natural and locally available materials as well as creating products that are sustainable. Undergirding all interactions with indigenous communities is the need for the recognition of trauma and misrepresentation that resulted from cultural imperialism and oppression, leading to a historical mistrust in the indigenous communities of outsider interventions. Talwar (2015) further highlights that issues around understanding multiculturalism can be meaningfully tackled within the context of historical representations of minority cultures within the culture in power. With this literature review we present a preliminary understanding of issues around working in indigenous settings and traditional materials that need further examination including implications for clinical practice and research approaches.

### Clinical Implications for Art Therapy

Art therapists interested in working with indigenous art forms or with indigenous communities need to first and foremost consider several aspects of their own identity and what they represent to indigenous communities. These include acknowledging personal motivations, power, and privilege; and willingness to reflect on personal history and its intersection with the colonial oppression, trauma, and multi-generational discrimination faced by most indigenous communities. Cultural awareness, knowledge, and humility are essential values in providing competent services, especially in culturally diverse indigenous settings. Given that there are now increasing numbers of art therapists working with people of diverse cultures both in their home country and abroad (Talwar, 2015), cultural competency (Sue, 2006) has been discussed in art therapy literature including in relation to making an effort and having an open attitude to learn the culture, being self-reflexive about one’s own biases and having a culturally humble stance to different values, beliefs, background (Ter Maat, 2011; Kapitan, 2015; Talwar, 2015; Potash et al., 2017). A related construct of cultural humility might serve art therapists well when working with traditional and indigenous artistic practices. Reflecting on one’s own cultural biases is a central value in the cultural humility framework. A culturally humble therapist examines their own cultural beliefs and identities and acknowledges their privileged position in the therapeutic relationship (Tervalon and Murray-Garcia, 1998). While being reflective and self-critical, the therapist should also be attentive to systemic forms of privilege and power in order to understand the oppressive mechanisms that can impact a client (Bal and Kaur, 2018). When therapists are perceived by the client as being respectful, interested, open and considerate, they are more likely to have better working alliances and improvements in therapeutic outcomes (Hook et al., 2013). This inclusive and culturally humble stance allows clinician to access the knowledge about culture without alienating the client (Bal and Kaur, 2018; Jackson, 2020). It is necessary for the art therapist to be familiar with the culture-specific media, technique and artistic philosophy. However, even when the art therapist feels competent in knowing the culture, the client is the expert on the culture of their specific community. Viewing the client as the expert and the teacher of their own culture validates and empowers the client to be more open when sharing their stories (Campanelli, 1996; Jackson, 2020). Art therapists might further explore and reflect on the artistic practices of their own heritage to better understand the meaning and health implications underlying creative practices.

Beyond the positioning of the art therapist, a key consideration in indigenous communities is the need to recognize that art making is often an integral part of the cultural system of the community, often with specific roles assigned by age and/or gender as well as valuing the interrelatedness of all things in nature (Campanelli, 1996). For example, traditional textiles including knitting, crocheting, weaving, embroidery, etc. tend to have historically be made by women but have also been found to forms of effective coping in the modern world (Collier, 2011). Other forms of woodwork, metalwork, fabric dying, etc. might be associated more with men’s roles and awareness of these traditional roles need to be recognized when interacting in the community (Kaimal et al., 2016). Kapitan (2015) discusses art therapy as a cultural practice, especially when working with marginalized cultures, acknowledging the risks of ethnocentric bias when we consider art making as a universal, non-verbal communication tool. In our review, we encountered studies and workshops where art therapists have tried to incorporate cultural practices such as the use of holistic healing practices. Such practices have included connecting an art therapy directive with existing artistic practices smudging, drumming and prayers in the therapy session (Lu and Yuen, 2012; Warson, 2012) with the goals of addressing specific health promoting goals in a non-hierarchical participatory way. When bringing art therapy into indigenous settings, art therapists need to co-create and work alongside a community mentor or leader in order to find culturally contextualized solutions to identified challenges in the problems in the community (Campanelli, 1996; Huss et al., 2015; Weinberg, 2018). Locating the meaning and value of art in the specific culture will allow art therapists to expand out of their own value systems and understand art making as a context-specific cultural phenomenon (Huss et al., 2015). The significance of culturally sensitive and relevant art therapy when working with indigenous clients was highlighted by several authors (Warson et al., 2013; Weinberg, 2018). The arts are an inherently valued source of community well-being (Campanelli, 1996) and in order to provide culturally relevant art therapy, the therapist needs to include the related learning norms and communication styles and avoid cultural misunderstandings (Ter Maat, 2011).

In addition to art therapists, artists, therapists, and healers have been practicing community healing programs that integrates traditional arts. For example, Archibald et al. (2010) conducted a survey with 137 Aboriginal foundations to investigate the use of creative arts relative to healing with first nation, Inuit and Metis healing programs in Canada. The self-report data showed that the use of creative arts in the aboriginal healing programs can make a meaningful contribution to the self-development, feeling personal and cultural safety, well-being and improved social relations. Lindeman et al. (2017) comprehensive literature concluded that art programs for Aboriginal and Torres Strait Australians living with dementia play a vital role in maintaining culture and traditional practices. Studies such as these indicate that art making can play a role as a protective factor; it can strengthen the community ties and increase self-esteem, elevate mood and change perception of pain, which can be of tremendous benefit for the indigenous communities (de Guzman et al., 2010; Muirhead and De Leeuw, 2013). Although these programs are not art therapy, they suggest positive health outcomes.

Incorporating traditional knowledge not only helps the indigenous community receive better care, but it may also offer therapeutic benefits to wider populations. Depending on the context, the use of culture-specific materials and forms of expression may be a better choice instead of conventional art therapy media. However, when bringing traditional art forms and materials outside of the cultural context, it is important to consider the dangers of cultural misappropriation. Even if the art therapist is well-informed, sensitive and ethical in their adaptation of cultural media and techniques into the therapeutic practice, and, is from the same cultural background, clients may not feel safe if there are conflicting assumptions around what is a culturally appropriate media or technique. This can potentially construct a superficial understanding of culture and help maintain already existing stereotypes. As Napoli (2019) highlights an art form or material may be profoundly significant to the original culture but when it is removed from the cultural context, the original meaning may get lost or worse, misrepresented. However, this doesn’t mean art therapists shouldn’t incorporate culture-specific art. The therapist can take an approach of being a partner in co-creating wellness, recognizing the interrelationships of all aspects of the community in creative practices, modeling respect and humility to counter the historic mistrust of indigenous communities toward the Western approaches to healthcare (Campanelli, 1996; Weinberg, 2018).

### Suggestions for Future Research

Throughout this paper, we have highlighted the potential ethical considerations of incorporating traditional knowledge and creative practices into the profession of art therapy. However, the literature review suggests a pronounced need for further research especially on culturally responsive methodologies. In particular the wisdom on traditional approaches needs to be unpacked in order to better understand the underlying knowledge systems. Traditional and indigenous knowledge systems are founded on generations of observations a deep understanding of culture and context specific needs health needs. These values are often at odds with the individually focused health ideals of modern Western medicine and therapies. Clinicians can identify which community characteristics play a role in leading to therapeutic outcomes by comparing intercultural responses to adapting certain traditional knowledge into art therapy. For instance, incorporating holistic knowledge into a practicing monotheistic community may require different considerations than incorporating holistic knowledge into communities which religion may have a belief systems. Working across disciplinary boundaries with artists and traditional healers would also be an area of further inquiry for art therapy researchers.

Given historical experiences of oppression and justifiable mistrust of external entities, appropriate methods are a major consideration as cautioned by Warson (2012) and Weinberg (2018). Using storytelling traditions, reflective and participatory methods, recognizing appropriate community-led engagement are essential to research in this topic. In addition specific approaches and questions might require age and gender related adaptations to align with the values of the community. Moreover, future research in art therapy and indigenous communities can benefit from methodologies that accurately and appropriately measure outcomes. Outcome measures such as standardized tests should be either adapted to the specific culture or replaced with other culturally acceptable data collection tools. Understanding the interplay between individual and community well-being and how artmaking effectively helps promote this intergration is an area for further study. Researchers interested in this work, need to begin first by reflecting on personal biases, exploring the challenges and concerns around misappropriation and cultural humility in integrating indigenous practices. Further research is needed to better understand whether and how to implement traditional artistic practices in art therapy including identifying ethical standards that are acceptable to the community for both engagement and research.

## Limitations and Conclusion

In this paper we provided an overview of the literature on the intersection of art therapy and indigenous and traditional artforms. The existing literature offers preliminary guidance but further research is needed to address the challenges of mistrust of individually focused Western approaches to health, while indigenous communities value interdependence as key to well-being. The solutions lie in the artistic practices themselves since indigenous art media and techniques can offer cultural safety and acknowledgment for the local communities. In addition art therapist may consider revisiting cultural humility framework, recognize the historical trauma and disenfranchisement experienced by many indigenous communities and approach their work through a participatory model of co-creation.

## Author Contributions

GK contributed to the overall framework for the manuscript as well as discussion sections. AA contributed to the literature review and writing of the manuscript.

## Conflict of Interest

The authors declare that the research was conducted in the absence of any commercial or financial relationships that could be construed as a potential conflict of interest.
